# Inhibiting lysine 353 oxidation of GRP78 by a hypochlorous probe targeting endoplasmic reticulum promotes autophagy in cancer cells

**DOI:** 10.1038/s41419-019-2095-y

**Published:** 2019-11-12

**Authors:** Junya Ning, Zhaomin Lin, Xuan Zhao, Baoxiang Zhao, Junying Miao

**Affiliations:** 1 Shandong Provincial Key Laboratory of Animal Cells and Developmental Biology, School of Life Science, Shandong University, Qingdao, 266237 PR China; 2 Central Research Laboratory, the Second Hospital, Shandong University, Jinan, 250033 PR China; 3 Institute of Organic Chemistry, School of Chemistry and Chemical Engineering, Shandong University, Jinan, 250100 PR China; 4 The Key Laboratory of Cardiovascular Remodeling and Function Research, Chinese Ministry of Education and Chinese Ministry of Health, Shandong University Qilu Hospital, Jinan, 250012 PR China

**Keywords:** Macroautophagy, Macroautophagy, Cell signalling, Cell signalling, Post-translational modifications

## Abstract

The level of hypochlorous acid (HOCl) in cancer cells is higher than that in non-cancer cells. HOCl is an essential signal for the regulation of cell fate and works mainly through the protein post-translational modifications in cancer cells. However, the mechanism of HOCl regulating autophagy has not been clarified. Here we reported that a HOCl probe named ZBM-H targeted endoplasmic reticulum and induced an intact autophagy flux in lung cancer cells. Furthermore, ZBM-H promoted the binding of GRP78 to AMPK and increased the phosphorylation of AMPK in a dose- and time-dependent manner. GRP78 knockdown inhibited ZBM-H-induced AMPK phosphorylation and ZBM-H-stimulated autophagy. In addition, mass spectrometry combined with point mutation experiments revealed that ZBM-H increased GRP78 activity by inhibiting HOCl-induced lysine 353 oxidation of GRP78. Following ZBM-H treatment in vitro and in vivo, cell growth was significantly inhibited while apoptosis was induced. Nevertheless, exogenous HOCl partially reversed ZBM-H-inhibited cell growth and ZBM-H-induced GRP78 activation. In brief, we found that an endoplasmic reticulum-targeted HOCl probe named ZBM-H, acting through attenuating HOCl-induced GRP78 oxidation, inhibited tumor cell survival by promoting autophagy and apoptosis. Overall, these data demonstrated a novel mechanism of hypochlorous acid regulating autophagy by promoting the oxidation modification of GRP78.

## Introduction

Hypochlorous acid (HOCl) is a well-known physiological oxidant that functions as a potent antimicrobial agent. HOCl is generated enzymatically by myeloperoxidase (MPO), a heme peroxidase released by activated neutrophils, with H_2_O_2_ and chloride anions as substrates^1^. In addition to its antimicrobial properties, HOCl is also an important signaling molecule for cell growth and regulates a range of physiological processes^2,3^. However, excessive hypochlorous acid contributes to a variety of diseases such as cancers, atherosclerosis, rheumatoid arthritis, inflammatory diseases and many others^4,5^. It has been reported that HOCl derived from neutrophils can promote DNA damage and mutation in lung epithelial cells, leading to lung cancer initiation^6^. However, the effector molecules involved in the regulation of lung cancer cell growth by HOCl are still unclear. Therefore, screening reliable small molecules that target hypochlorous acid is very significant for elucidating HOCl’s physiological functions. It has been reported that the mode of action of HOCl regulating physiological processes is through the post-translational modifications of proteins^2,3,5^. Oxidation and chlorination modification of proteins caused by HOCl leads to changes in proteins activity^1^. The function of exogenous HOCl-modified proteins has been studied in great detail^1,7–9^. However, the mechanism by which endogenous HOCl modifies proteins and the mechanism of HOCl-modified proteins regulating tumor cell growth have not been clarified.

Glucose-regulated protein 78 (GRP78), as one of the best-characterized endoplasmic reticulum (ER) chaperone proteins, regulates the folding and transport of newly synthesized proteins and is also involved in reducing the protein folding load in ER to maintain cell function^10^. It has been shown that GRP78 expression levels were positively correlated with human lung adenocarcinoma cell apoptosis and chemosensitivity of a novel gibberellin derivative^11^. However, whether GRP78 activity is related to lung cancer cell apoptosis and autophagy has not been clarified^12^. A recent study has suggested that glutathionylation of a cysteine in the molecular chaperone GRP78 in yeast promoted cell survival by inhibiting peroxide-induced irreversible oxidation under oxidative stress^13^. Oxidized non-selenocysteine containing phospholipids hydroperoxide glutathione peroxidase (NPGPx) has been reported to binds and oxidizes GRP78 through covalent bonding between Cys86 of NPGPx and Cys41/Cys420 of GRP78, which facilitates the refolding of misfolded proteins by GRP78 to alleviate stress^14^. The data suggest that post-translational modifications, especially oxidation modifications, play a vital role in controlling GRP78 activity. However, it is still unclear how HOCl affects oxidation modification of GRP78 and its activity in cancer cells.

Autophagy is a highly regulated biological process in which double-membrane autophagosomes sequestered cytoplasmic macromolecules and organelles and then fused with lysosomes for degradation and recycling. And autophagy is also involved in the regulation of cell growth^15^. Recent studies have reported that autophagy had potential anti-tumor properties^16–18^. Apoptosis, also known as programmed cell death, has been shown to be a major type of cell death to suppress the progress of cancer^19^. Although HOCl is clearly involved in the regulation of apoptosis, its role in autophagy has rarely been studied^20^. Likewise, it remains unknown whether HOCl affects apoptosis and autophagy by altering the post-translational modification of GRP78.

Emerging evidence suggests that HOCl plays an important role in regulating tumor growth because HOCl production is higher in cancer cell lines than that in non-cancer cell lines^21^. Many studies have focused on developing new tools that can detect HOCl in different organelles^22,23^. However, the application of them to explore the mechanism of HOCl-regulated cell growth is rarely studied. In the current study, we aimed to explore potential effector molecules involved in HOCl regulating cell growth by taking advantage of small chemical molecules, which affect cell fate via targeting HOCl and interfering with its function, thereby providing new evidence for the function of endogenous HOCl-modified proteins in cancer. In a previous study we synthesized and identified a new ratiometric fluorescent probe (ZBM-H) based on benzimidazole-hemicyanine for selectively detecting endogenous hypochlorous acid in living cells with high sensitivity^24^. But it is unclear whether ZBM-H regulates cancer cell fate by affecting the post-translational modifications of proteins induced by endogenous HOCl. We now report that ZBM-H promotes autophagy and apoptosis in cancer cells through inhibiting HOCl-caused oxidation modification of GRP78.

## Materials and methods

### Chemicals

BafiIomycinA1 (BafA1) was purchased from Sigma Aldrich, The United States. Compound C was purchased from Selleck, The United States.

### Antibodies

Antibodies for LC3B (2775 S), AMPK α subunit (AMPKα) (5832 T), p-AMPKα (2535 T), p-4EBP1 (2855 S) and 4EBP1 (7452 S) were from Cell Signaling Technology (CST), The United States. SQSTM1 (PM045) antibody was from MBL. β-actin (A5441) was from Sigma Aldrich. AMPKα (sc-398861) antibody was from Santa Cruz Biotechnology, The United States. Antibodies for GRP78 (11587-1-AP) and His (66005) were from Proteintech Group, China. Horseradish peroxidase-conjugated secondary antibodies were from Jackson immunoresearch. Secondary antibodies for immunofluorescence were donkey anti-rabbit IgG Alexa Fluor-546 (A-11037; Invitrogen), donkey anti-mouse IgG Alexa Fluor-546 (A10036; Invitrogen) and donkey anti-rabbit IgG Alexa Fluor-633 (A32795; Invitrogen).

### Cell culture

All the cells utilized in the experiment were purchased from the Cell Culture Bank of the Chinese Academy of Sciences (http://www.cellbank.org.cn/). Human lung cancer cell line A549 cells were cultured in RPMI-1640 medium supplemented with 10% (v/v) bovine calf serum. U87 cells, which were stably transfected with GFP-LC3B, HEK293 cells and Hela cells were cultured in Dulbecco’s modified Eagle’s medium (DMEM, Gibco, 12800-058) with 10% fetal bovine serum. Human umbilical vein endothelial cells (HUVEC) were grown in M199 medium (Gibco, 31100-035) with 10% (v/v) bovine calf serum and 8.4 IU/mL FGF2. All cell lines were cultured in a humidified incubator at 37 °C under 5% CO_2_ atmosphere. The cells were seeded onto appropriate dishes (35,000 cells/ml). All cell lines were authenticated by DNA short tandem repeat (STR) profiling and confirmed to be mycoplasma negative.

### Cell morphology

Morphologic changes of A549 cells treated with ZBM-H at indicated concentration for 3, 6, 12, 24, and 48 h were examined by inverted phase-contrast microscope (Eclipse TS-100; Nikon, Tokyo).

### Cell viability assay

A549 cells were seeded onto 96-well plates, and then treated with 0.1% DMSO (as control) or ZBM-H at indicated concentration for 24 h. Cell viability was determined by sulforhodamine B (SRB) assay according to the manufacturer’s instructions.

### Western blot analysis

Briefly, cell lysates were applied to SDS-polyacrylamide gel and electroblotted to poly-vinylidene difluoride (PVDF) membranes (Millipore, USA). At room temperature, the membrane was blocked with 5% non-fat milk in TBST (TBS containing 0.05% Tween-20) for 1 h. Thereafter, the membrane was incubated with primary antibody overnight at 4 °C and was then washed 3 times with TBST, each time for 5 min. The membrane was subsequently incubated with corresponding secondary antibodies that are Horseradish peroxidase-conjugated for 1 h at room temperature and was then washed 3 times with TBST, each time for 5 min. And proteins were detected by use of an enhanced chemiluminesence detection kit (Thermo Fisher, 34080). The relative quantity of proteins was analyzed by Image J software and normalized to loading controls.

### Evaluation of fluorescent LC3 puncta

The pmCherry-GFP-LC3B plasmid was purchased from MiaoLingBio. Briefly, A549 cells were transfected with mCherry-GFP-LC3B for 24 h, and then treated with indicated compounds. After 6 h, cells were fixed, stained with DAPI and LC3B puncta (yellow dots and free red dots) was analyzed by manual counting of fluorescent puncta in five fields from three independent experiments with 50 cells/field^25,26^.

### Immunofluorescence Assay

Treated cells were fixed in 4% paraformaldehyde (w/v) for 30 min at room temperature and then incubated with normal donkey serum (1:30) for 30 min and primary antibodies (1:100) overnight at 4 °C. Cells were washed with phosphate buffered saline (PBS) 3 times, and then incubated with secondary antibodies (1:200) for 1 h at 37 °C. Fluorescence was detected by laser scanning confocal microscopy Zeiss LSM700 (Germany). Frozen sections of tumors formed on the chick embryo chorioallantoic membrane (CAM) were fixed with cold acetone for 10 min and blocked with 10% normal donkey serum (Solarbio, SL050) for 30 min at room temperature. Then frozen sections of tumors were incubated with primary antibody (1:100; LC3B, Rabbit polyclonal antibody, Santa Cruz Biotechnology) at 4 °C overnight and then corresponding secondary antibody (1:200) at 37 °C for 1 h. Frozen sections of tumors were washed 3 times with 0.1 M PBST. DAPI (1:200) was added to stain cell nucleus for 10 min and then the sections were washed 3 times with PBS. Fluorescence was detected by confocal fluorescence microscopy Zeiss LSM700 (Germany).

### Immunoprecipitation (IP)

A549 cell lysates were pre-cleared with protein A/G agarose beads (Beyotime, P2012) for 1 h at 4 °C. After centrifugation, the supernatant was collected and incubated with specific antibodies or normal corresponding IgG, then with protein A/G beads overnight at 4 °C. The beads were washed with IP buffer 3 times and eluted with 2 × SDS loading buffer. The immunoprecipitated proteins were detected by western blot assay.

### RNA interference

Specific siRNA against GRP78 was designed and custom-synthesized by Boshang. A549 cells at 70–80% confluence were transfected with 60 nM siRNA against GRP78 and scramble siRNA with Lipofectamine 2000 (Invitrogen, 11668–019) according to the manufacturer’s instructions. Then cells were harvested and analyzed by western blot assay.

### Plasmids and overexpression

We cloned full-length GRP78 into the pENTER plasmid (pENTER-GRP78). His-tagged GRP78 plasmids including his_6_-GRP78-wt (wild type), his_6_-GRP78-mut1 (K352A) (mutant) and his_6_-GRP78-mut2 (K353A) (mutant) were constructed first. A549 cells at 70–80% confluence were transfected with the expression vectors for 24 h using Lipofectamine 2000 (Invitrogen, 11668–019) according to the manufacturer’s instructions. Then cells were harvested and analyzed by western blot assay.

### Mass spectrometric analysis

Briefly, purified GRP78 protein was digested in-solution by trypsin, and then analyzed by liquid chromatograph tandem mass spectrometry (LC–MS/MS) using a Q Exactive mass spectrometer (Thermo Scientific) that was coupled to Easy nLC (Thermo Fisher Scientific). The mass spectrometer was operated in positive ion mode. MS1 scans were acquired using a mass resolution of 70,000, and MS2 scans were obtained with a resolution of 17,500. The raw data from mass spectrometry was searched against database using Mascot. Lysine oxidation products (C_6_H_11_NO_4_) were included in the search.

### In vitro GRP78 activity assay

HEK293 cells were seeded onto 10-cm dishes and then transfected with the indicated expression vector including his_6_-GRP78-wt (wild type), his_6_-GRP78-mut1 (K352A) (mutant) and his_6_-GRP78-mut2 (K353A) (mutant) after the density of HEK293 cells reached 50–60% confluence. After 48 h, total proteins were harvested. And expressed recombinant GRP78 was extracted and purified by Ni-NTA protein purification assay. Purified GRP78 was incubated at 37 °C with or without ZBM-H for indicated concentrations and times. The ATPase activity of GRP78 was measured by using the ATPase/GTPase Activity Assay Kit (MAK113, Sigma, USA).

### Hoechst 33258 staining

A549 lung cancer cells treated with DMSO or ZBM-H were stained with Hoechst 33258 at 10 mg/mL and incubated at 37 °C for 15 min. Cells were washed with PBS twice and then observed by using an Olympus (Japan) BH-2fluorescence microscope. Apoptotic cells were identified by intense local staining of condensed DNA, with diffuse DNA staining in normal cells. At least 300 cells were counted for this assay.

### Terminal deoxynucleotidyl transferase-mediated dUTP nick-end labeling (TUNEL)

Frozen sections of tumors formed on the chick embryo chorioallantoic membrane (CAM) were fixed with cold acetone for 10 min. DNA fragmentation of treated tumor tissues was detected by using the Dead End TM Fluorometric TUNEL System (Roche) according to the manufacturer’s instructions, wherein laser scanning confocal microscopy (Leica, Wetzlar, Germany) was used. The excitation wavelength used in this assay was 555 nm.

### In vivo tumor assay of chick embryo chorioallantoic membrane (CAM)

The fertilized chicken eggs were incubated at 37 °C with 60% relative humidity. On embryonic day 8, eight million A549 lung cancer cells in 20 μl of medium were seeded into the silicone ring, a silicone ring of 5.5 mm inner diameter placed on the CAM. And then eggs were divided into three groups, per group containing 5 eggs. After 2 days, the eggs were treated with ZBM-H at the concentrations of 50 and 100 μM for every 2 days, with DMSO as the negative control group. After treatment with ZBM-H for 3 times, the CAM and tumors were collected. The tumors were photographed and the tumor volume calculation was performed as described in the literature^27^.

### Angiogenesis assay of CAM

The fertilized chicken eggs were incubated at 37 °C with 60% relative humidity. On embryonic day 9, the CAMs were treated with ZBM-H (50 or 100 μM) or DMSO soaked in the gelatin sponge. The CAMs were treated with ZBM-H and DMSO for every 2 days. After 6 days, the treated CAM were sampled and photographed. Angiogenesis was analyzed with Image-Pro Plus as previously described^28^.

### Statistical analysis

Data were presented as means ± SE from at least three separate experiments and analyzed by t-test with SPSS 17.0 (SPSS Inc., Chicago, IL, USA). Differences with a *p* < 0.05 were recognized as statistically significant.

## Results

### ZBM-H targeted endoplasmic reticulum (ER)

**Fig. 1:**
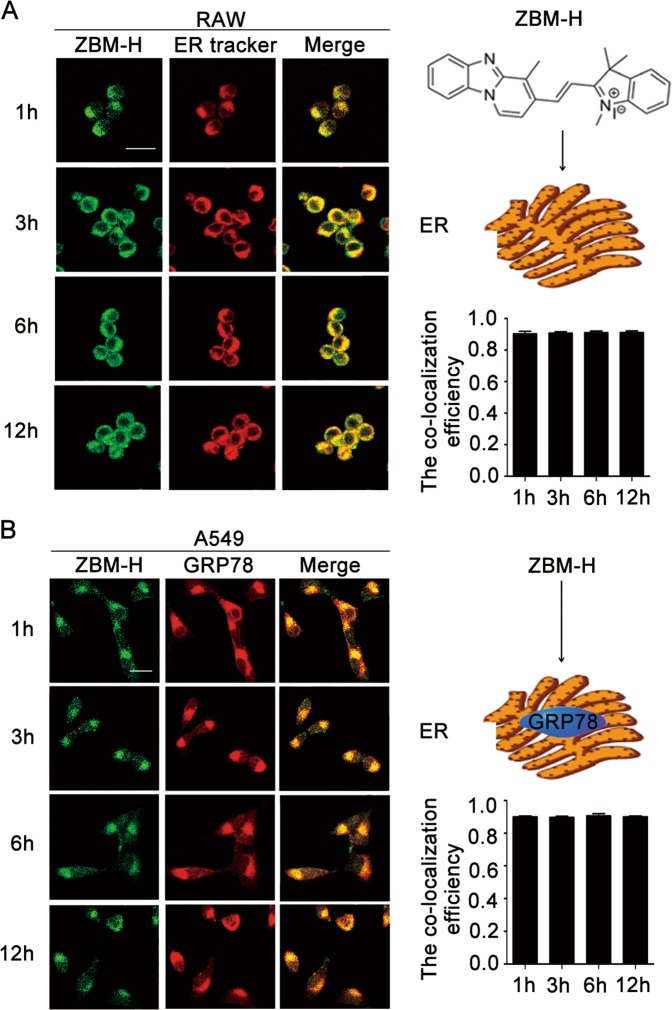
ZBM-H targeted endoplasmic reticulum. **a** The co-localization efficiency of ZBM-H and endoplasmic reticulum (ER) was 0.91 in RAW cells treated with ZBM-H (2 μM) and ER Tracker for 1, 3, 6, 12 h. **b** Immunofluorescence (IF) staining of GRP78 (excitation light was 546 nm) in A549 cells treated with ZBM-H (2 μM) for 1, 3, 6, 12 h, and the co-localization efficiency of ZBM-H and GRP78 was 0.90. Scale bar: 20 μm

Small chemical molecules targeting HOCl in different organelles had varied effects on cell fate according to our data (Fig. S1 and Table S1-S3). In order to determine the distribution of HOCl probe (ZBM-H) in the organelles, we firstly investigated the organelle targeting of ZBM-H taking advantage of its good fluorescence characteristics. Co-localization analysis of mitotracker with ZBM-H revealed that ZBM-H had no co-localization with mitochondria (Fig. S2). Interestingly, we found that ZBM-H had good co-localization with ER tracker (Fig. 1a). In addition, we performed immunofluorescence staining and further determined that ZBM-H surely targeted endoplasmic reticulum, as indicated by good co-localization of ZBM-H with GRP78, a classical ER marker (Fig. 1b).

### Autophagy was induced in A549 lung cancer cells treated with ZBM-H

**Fig. 2:**
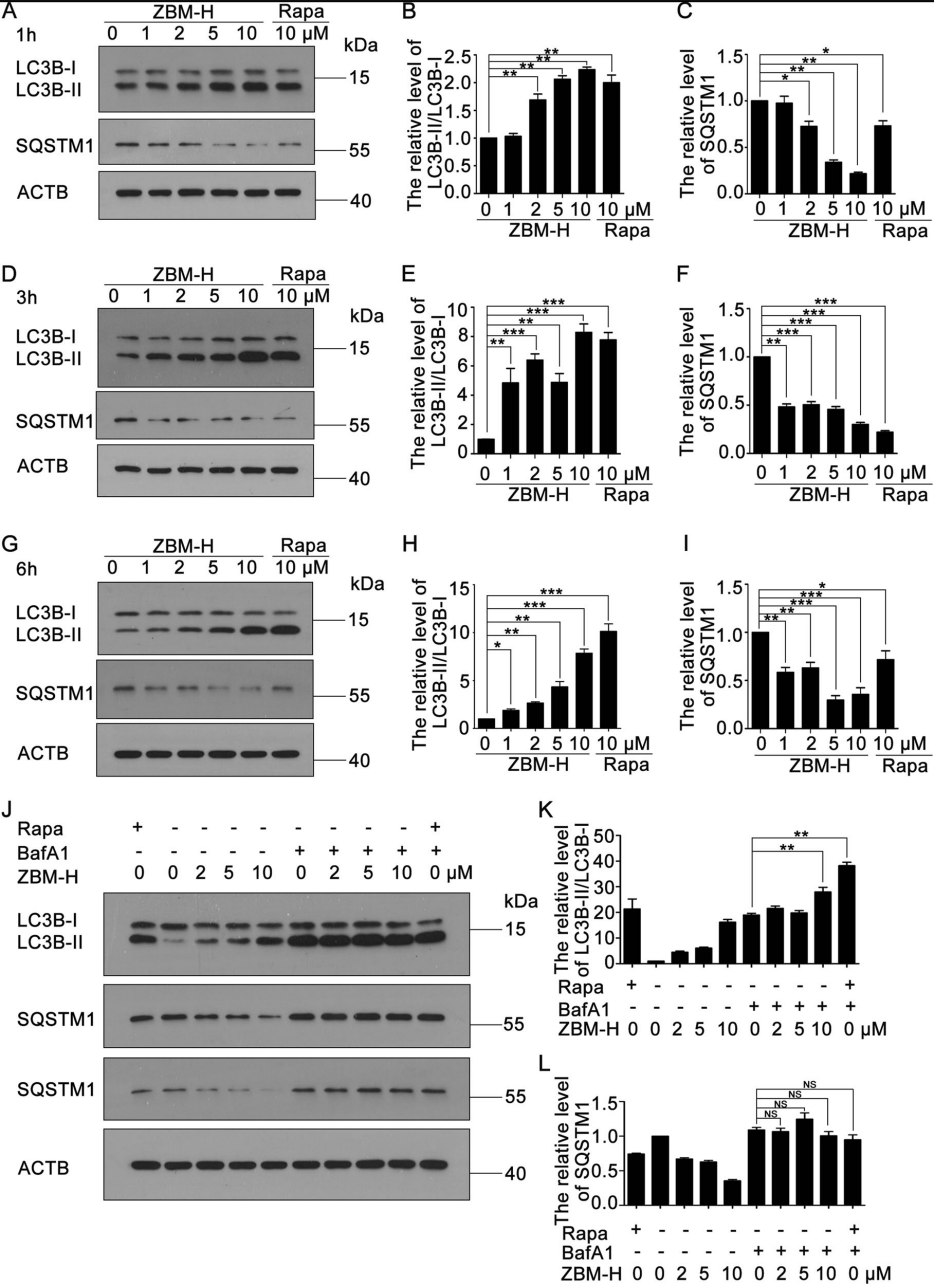
Autophagy was activated in A549 lung cancer cells treated with ZBM-H. A549 cells were treated with ZBM-H for 1 h **a**–**c**, 3 h **d**–**f**, and 6 h **g**–**i** the protein levels of LC3B-II/LC3B-I and SQSTM1 were determined by Western Blot. Rapamycin (Rapa) was used as a positive control. **j**–**l** Western Blot analysis of LC3B-II/LC3B-I and SQSTM1 in cells treated with BafA1 (50 nM) and ZBM-H for 6 h, with Rapa (10 μM) as positive control. Data are presented as the mean ± SEM, *p *<* 0.05, **p *<* 0.01, ***p *<* *0.001, n* *=* *3*

**Fig. 3:**
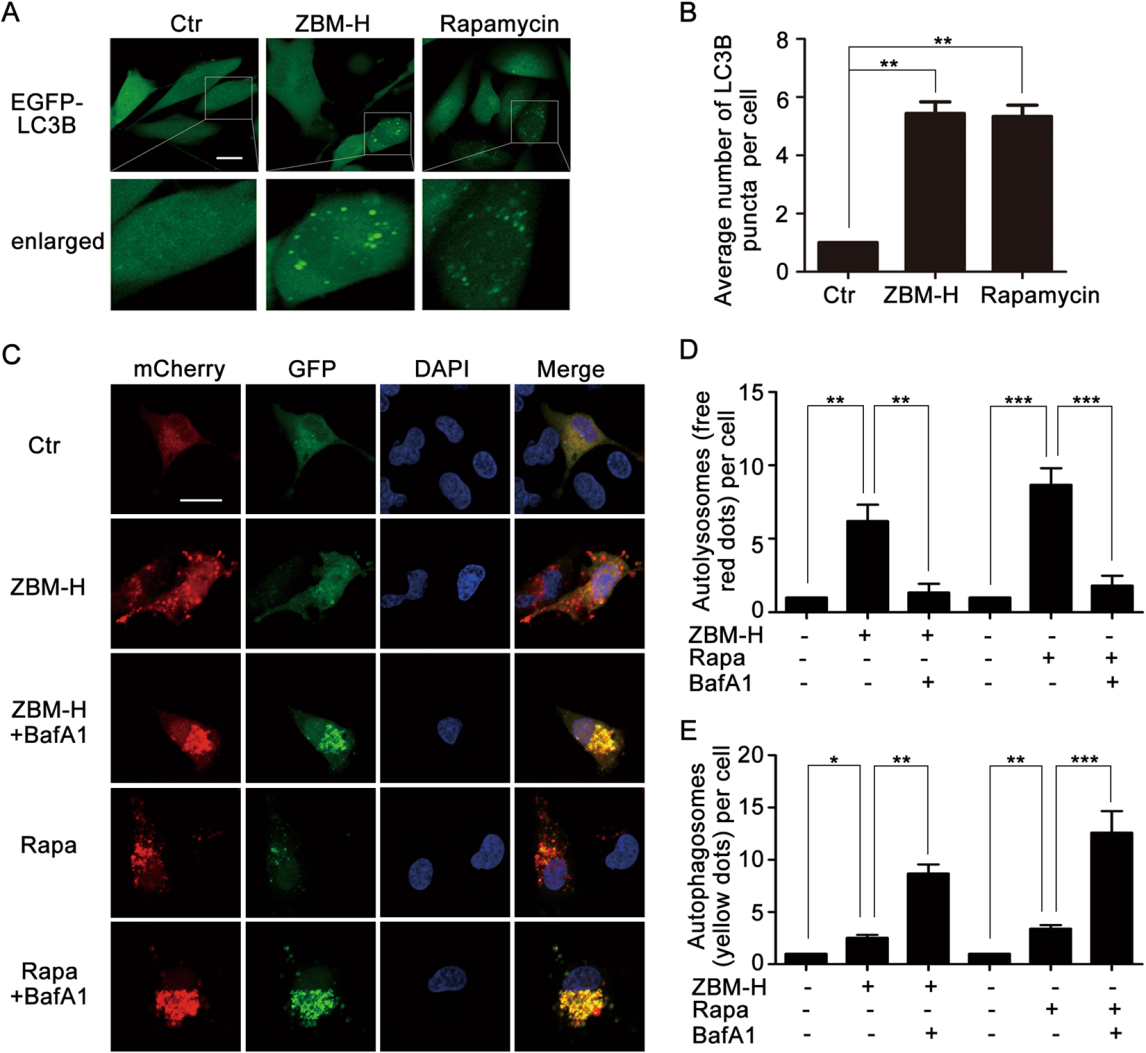
ZBM-H promoted autophagy flux in A549 cells. **a**, **b** MAP1LC3B puncta was analyzed in U87 cells treated with ZBM-H (2 μM) and Rapamycin (2 μM) for 6 h. Scale bar: 20 μm. **c**–**e** A549 cells were transfected with mCherry-GFP-LC3B for 24 h, followed by treatment with ZBM-H, Rapamycin and Baf-A1. The fluorescence signals were visualized by confocal microscopy. Scale bar: 20 μm. Data are presented as the mean ± SEM, *p *<* *0.05,* **p *<* *0.01,* ***p *<* *0.001*

Recently, growing evidence has shown that autophagy plays a role in tumor-suppressing^29^. To examine whether ZBM-H regulated autophagy in lung cancer cells, we firstly determined how ZBM-H affected the formation of the autophagosome and the distribution of microtubule associated protein 1 light chain 3 beta (LC3B), which were essential for the autophagy process^30^. Indeed, ZBM-H treatment evidently increased the protein level of LC3B-II (Fig. 2a, b, d, e and g, h), which plays an essential role in the extension of the autophagosome. In addition, cells treated with ZBM-H also exhibited decreased Sequestosome-1 (SQSTM1) (Fig. 2c, f and i), a classical autophagy substrate. To further explore whether ZBM-H can induce intact autophagy flux, we examined the level of LC3B-II/LC3B-I and SQSTM1 in cells treated with ZBM-H and BafiIomycinA1 (BafA1), a compound blocking autophagy by altering the pH of lysosome, combined. We found that ZBM-H resulted in a further increase of LC3B-II/LC3B-I level in the presence of BafA1, while the level of SQSTM1 had no significant changes (Fig. 2j–l). Stable cell lines expressing GFP-LC3B are sensitive to autophagy inducers and show increased numbers of cytoplasmic GFP-LC3B puncta upon autophagic stimuli^30^. When U87 cells stably transfected with GFP-LC3B were treated with ZBM-H, we observed remarkable LC3B puncta, the classical marker of autophagy (Fig. 3a, b). The mCherry-GFP-LC3B plasmid is an efficient tool for detecting autophagy flux^30^. The signal of green (GFP) is quenched by the low pH inside the lysosome lumen, whereas the red signal (mCherry) exhibits more stable fluorescence in acidic conditions^30,31^. Data revealed that ZBM-H treatment increased formation of autophagosomes (yellow dots) and autolysosomes (free red dots) in cells transfected with mCherry-GFP-LC3B plasmid, which was similar to rapamycin treatment (Fig. 3c–e). Combinatorial treatment of BafA1 (a specific inhibitor of fusion between autophagosomes and lysosomes) and ZBM-H resulted in further accumulation of autophagosomes (yellow dots), while autolysosomes (free red dots) dramatically reduced (Fig. 3c–e). Taken together, these data indicated that ZBM-H not only increased autophagosome formation, but also enhanced autophagy flux in A549 lung cancer cells.

### GRP78/AMPK/mTOR pathway was involved in ZBM-H-induced autophagy

**Fig. 4:**
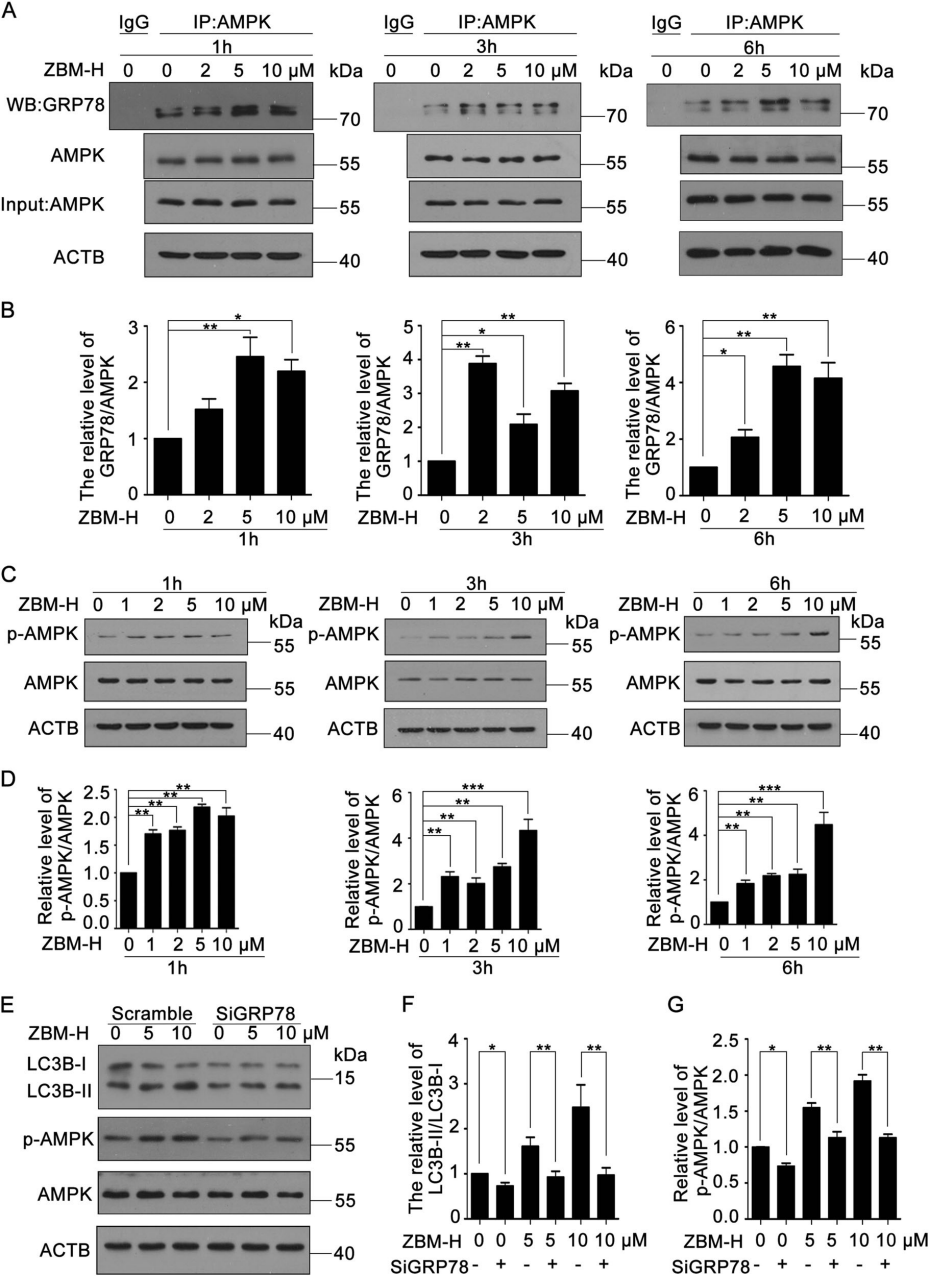
GRP78 was required for ZBM-H-promoted AMPK phosphorylation and autophagy. **a**, **b** Interaction between GRP78 and AMPKα was determined by co-IP assay in A549 cells treated with ZBM-H at indicated concentrations for 1 h, 3 h and 6 h. **c**, **d** Western blot analysis of phosphorylation of AMPKα (p-AMPKα) and AMPKα in A549 cells treated with ZBM-H at indicated concentrations for 1 h, 3 h and 6 h. **e**–**g** Western Blot analysis and quantification of LC3B-II/LC3B-I and phosphorylation of AMPKα (p-AMPKα) in A549 cells transfected with GRP78 siRNA (siGRP78 concentration was 60 nM) and then treated with ZBM-H (5, 10 μM) for 6 h. Data are presented as the mean ± SEM, *p *<* 0.05, **p *<* 0.01, ***p *<* *0.001, n* *=* *3*

GRP78 is essential for autophagy regulation^32^. A previous study has shown that overexpression of GRP78 activated 5ʹ-adenosine monophosphate (AMP)-activated protein kinase (AMPK)^33^. In order to explore the role of GRP78 in ZBM-H-induced autophagy, we first analyzed the interaction between GRP78 and AMPK by co-Immunoprecipitation (co-IP) and found that ZBM-H further promoted the interaction between GRP78 and AMPK in a dose- and time-dependent manner (Fig. 4a, b). Consistently, immunofluorescence assay revealed that ZBM-H facilitated the co-localization of these two proteins (Fig. S3), thereby increasing the phosphorylation of AMPK (Fig. 4c, d). However, Compound C, a physical inhibitor of AMPK, surely suppressed ZBM-H-induced autophagy (Fig. S4A-S4C). And 3-benzyl-5-((2-nitrophenoxy) methyl)-dihydrofuran-2(3 H)-one (3BDO), an activator of mechanistic target of rapamycin kinase complex 1 (mTORC1)^34^, also inhibited ZBM-H-induced autophagy by detecting the level of LC3B-II (Fig. S4D-S4E). Data also showed that ZBM-H treatment suppressed mTORC1 activity, as illustrated by decreased phosphorylation levels of ribosome S6 protein kinase (p70S6K) and eukaryotic translation initiation factor 4E binding protein 1 (EIF4EBP1/4EBP1), two essential substrates of mTORC1 (Fig. S4F-S4J).

In addition, knockdown of GRP78 with specific siRNA significantly reduced phosphorylation of AMPK and autophagy induced by ZBM-H (Fig. 4e–g and Fig. S5). These data suggested that GRP78/AMPK/mTOR pathway was involved in ZBM-H-induced autophagy in lung cancer cells.

### ZBM-H enhanced GRP78 activity via inhibiting HOCl-induced lysine 353 oxidation modification of GRP78

**Fig. 5:**
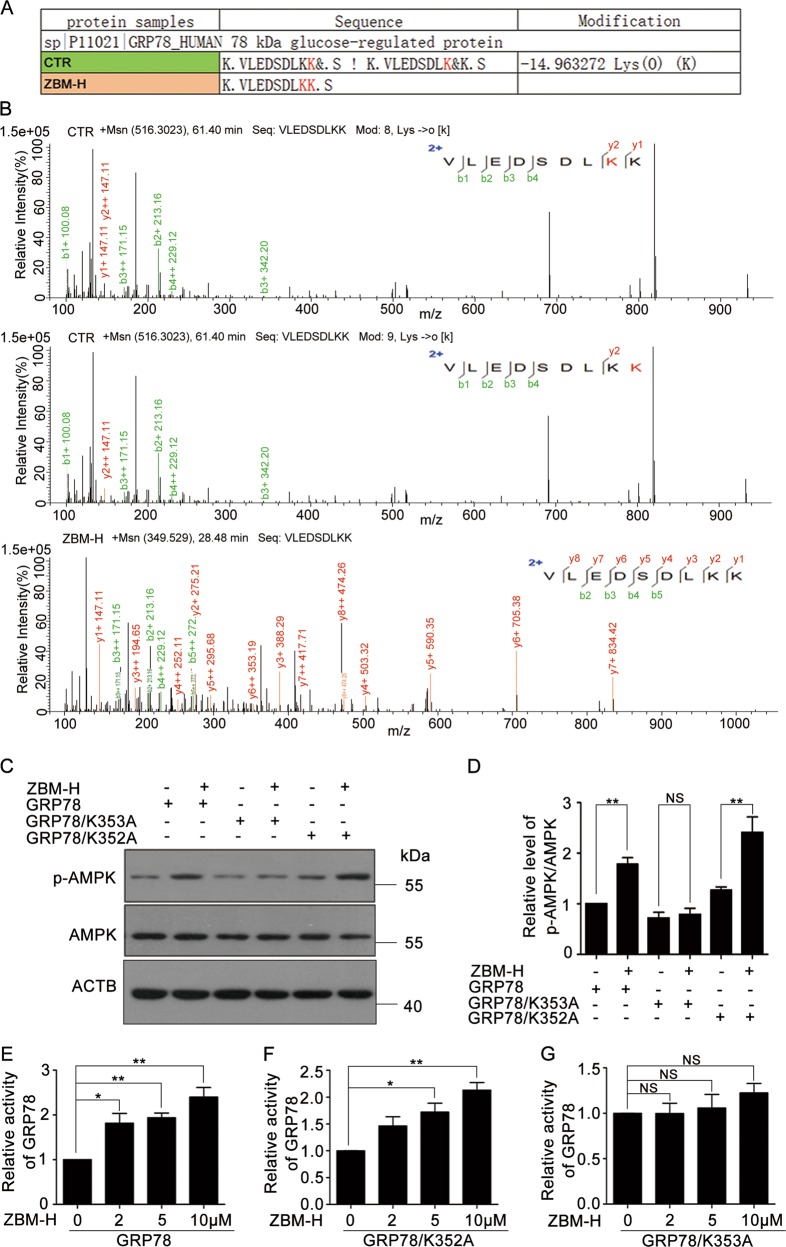
ZBM-H inhibited oxidation of GRP78 caused by HOCl to enhance its activity. **a** Mass spectrometry analysis showed that ZBM-H inhibited GRP78 oxidation modification at lysine 352 and 353. **b** Example of MS/MS spectra of the lysine-oxidized peptide VLEDSDLK[Oxi]K[Oxi]. The tryptic digest of purified GRP78 from cells treated with ZBM-H or DMSO was analyzed with the use of LC–MS/MS. **c**, **d** Western blot analysis of phosphorylation of AMPKα (Thr172) in A549 cells transfected with his_6_-GRP78-wt (wild type), his_6_-GRP78-mut2 (K353A) mutant and his_6_-GRP78-mut1 (K352A) mutant plasmids and treated with ZBM-H. **e**–**g** ATPase activity of GRP78-wt, GRP78-mut1 and GRP78-mut2 incubated with ZBM-H at indicated concentrations. Data are presented as the mean ± SEM, *NS p* *>* 0.05, *p *<* 0.05, **p *<* *0.01, n* *=* *3*

Since ZBM-H targeted and bound to intracellular HOCl, we hypothesized that the regulation of GRP78 activity by ZBM-H was probably through post-translational modifications. To test this hypothesis, we analyzed GRP78 protein purified from cells treated with DMSO or ZBM-H using LC–MS/MS. LC-MS/MS analysis of the purified GRP78 from DMSO-treated cells revealed a modified peptide, ^345^VLEDSDLKK^353^, with a molecular weight changed by 14.9632 Da (Fig. 5a) relative to the mass of the native peptide. Subsequent fragmentation of the modified peptide and analysis of the resulting b and y ions (Fig. 5b) allowed identification of K352 and K353 as the modified residues. MS-based analysis of the purified GRP78 from ZBM-H-treated cells did not exhibit modification of the ^345^VLEDSDLKK^353^ peptide. This result suggested that ZBM-H was able to inhibit lysine 352 and lysine 353 oxidation modification of GRP78 induced by HOCl. To illustrate how oxidation modification of GRP78 affected its activity, we transfected his_6_-GRP78-wt (wild type), his_6_-GRP78-mut1 (K352A) (mutant) and his_6_-GRP78-mut2 (K353A) (mutant) plasmids into A549 cells and then treated cells with ZBM-H. Data revealed that ZBM-H increased the phosphorylation of AMPK in the presence of his_6_-GRP78-wt as well as his_6_-GRP78-mut1. However, ZBM-H was not capable of enhancing the phosphorylation of AMPK in the presence of his_6_-GRP78-mut2 (Fig. 5c, d). In addition, his_6_-GRP78-mut2 partially protected cells from death induced by ZBM-H (Fig. S6).

To further evaluate how oxidation modification of GRP78 affected its ATPase activity, we transfected his_6_-GRP78-wt (wild type), his_6_-GRP78-mut1 (K352A) (mutant) and his_6_-GRP78-mut2 (K353A) (mutant) plasmids into HEK293 cells for 48 h. Data revealed that ZBM-H dose- and time-dependently increased ATPase activity of GRP78 purified from HEK293 cells transfected with his_6_-GRP78-wt (wild type) (Fig. 5e and Fig. S7). Similarly, ZBM-H also increased the ATPase activity of his_6_-GRP78-mut1 (Fig. 5f). However, ZBM-H failed to increase the ATPase activity of his_6_-GRP78-mut2 (Fig. 5g), which was consistent with the previous data. Taken together, these data suggested that lysine 353 was the potential site in GRP78 that was modified by HOCl to regulate its activity.

### ZBM-H inhibited lung cancer growth and induced apoptosis

**Fig. 6:**
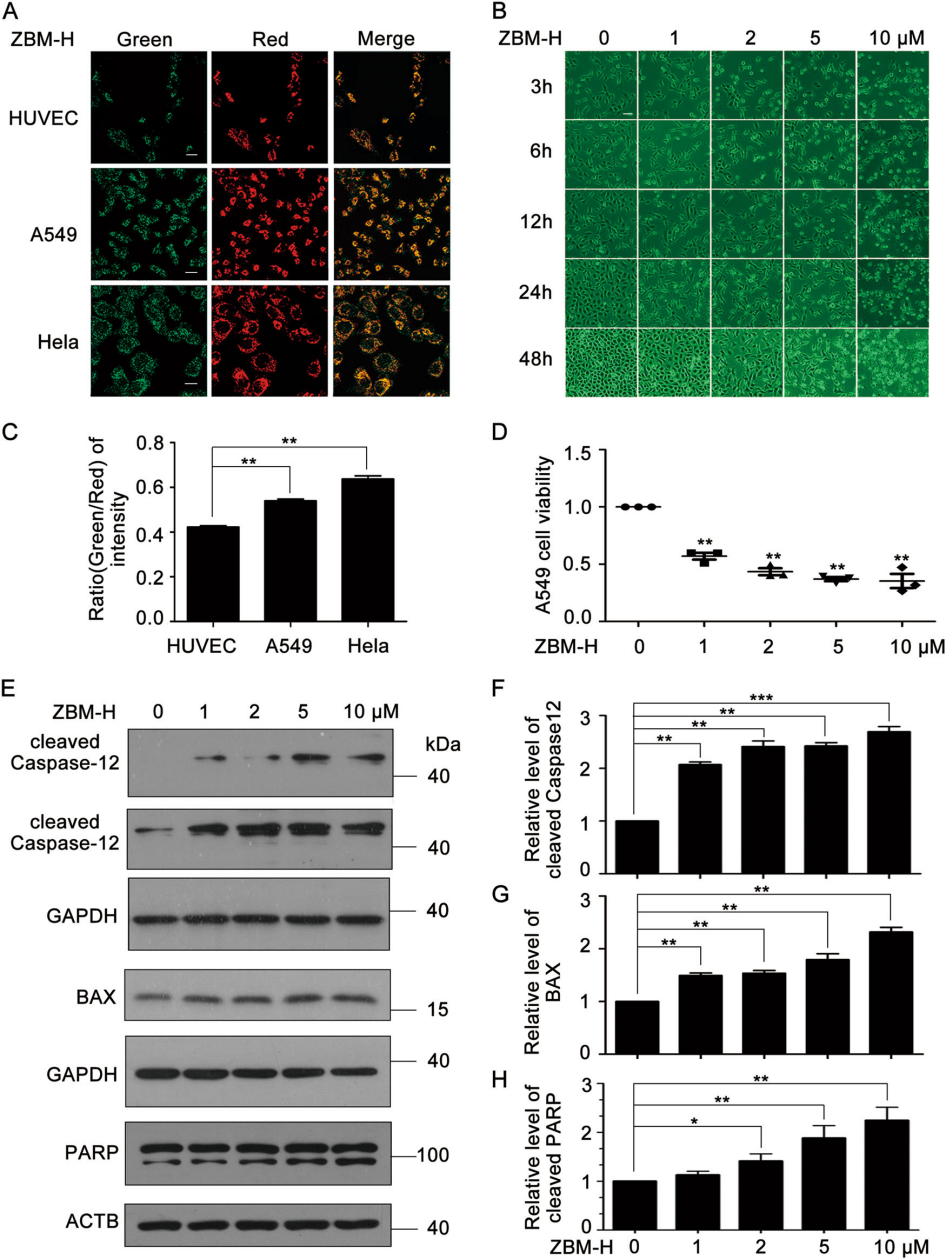
ZBM-H inhibited lung cancer cell growth and induced apoptosis. **a**, **c** Hypochlorous acid level determined in non-cancer cell and cancer cells. Human umbilical vein endothelial cells (HUVEC), A549 cells and Hela cells were incubated with ZBM-H (2 μM) for 30 min, and the change of fluorescence ratio was counted. Scale bar: 20 μm. **b** Morphological change of A549 cells treated with ZBM-H for indicated concentration. Scale bar: 20 μm. **d** A549 cell (treated with ZBM-H for 24 h) viability. **e**–**g** Western blot (WB) analysis of cleaved caspase-12 and BAX in A549 cells treated with ZBM-H at indicated concentrations for 24 h. glyceraldehyde-3-phosphate dehydrogenase (GAPDH) was used as loading control. **e**, **h** WB analysis of cleaved poly ADP-ribose polymerase (PARP) in A549 cells treated with ZBM-H for 24 h. Data are presented as the mean ± SEM, *p *<* 0.05, **p *<* 0.01, ***p *<* *0.001, n* *=* *3*

Emerging evidence suggests that HOCl plays an important role in regulating tumor growth as its production is higher in cancer cell lines than that in non-cancer cell lines^21^. In order to confirm whether the hypochlorous acid level in the tumor cells is high, we applied the ratiometric fluorescent probe named ZBM-H^24^ and performed the fluorescence imaging of ZBM-H in human umbilical vein endothelial cells (HUVEC), A549 cells and Hela cells. It was found that the corresponding ratio (green/red) of fluorescence intensity of A549 cells and Hela cells were higher than that of HUVEC, suggesting that hypochlorous acid level is higher in A549 cells and Hela cells than that in HUVEC (Fig. 6a, c). To explore the effect of ZBM-H on cancer cell growth, we firstly examined the morphological changes in A549 cells after treatment with ZBM-H at 1, 2, 5, and 10 μM for 3, 6, 12, 24 and 48 h using an inverted phase-contrast microscope. The living cell density declined dramatically in a time and concentration-dependent manner. And A549 cells significantly shrank and even changed to globular with a high concentration of ZBM-H treated (Fig. 6b). Furthermore, we investigated the cell viability of A549 lung cancer cells using sulforhodamine B (SRB) assay after treatment with ZBM-H at indicated concentrations for 24 h. Data revealed that ZBM-H significantly inhibited lung cancer cell survival, with a low IC_50_ value being 1.648 μM (Fig. 6d).

Apoptosis is one of the important processes of programmed cell death that is essential for cells to cope with the damage induced by various stressors and inhibits tumor growth effectively^35^. To detect the effect of ZBM-H on apoptosis, we performed a western blot experiment to measure the levels of apoptosis-associated protein. And we found that the protein level of cleaved caspase-12 and BAX increased (Fig. 6e–g), accompanied by increased cleavage of PARP (poly ADP-ribose polymerase) after treatment with ZBM-H at indicated concentrations (Fig. 6e, h). In addition, Hoechst 33258 staining revealed that ZBM-H notably augmented the apoptosis ratio of A549 lung cancer cells (Fig. S8). In summary, all these data revealed that ZBM-H effectively inhibit lung cancer growth and induced apoptosis.

### Exogenous hypochlorous acid partially reversed ZBM-H-inhibited cell growth and ZBM-H-increased GRP78 activity

**Fig. 7:**
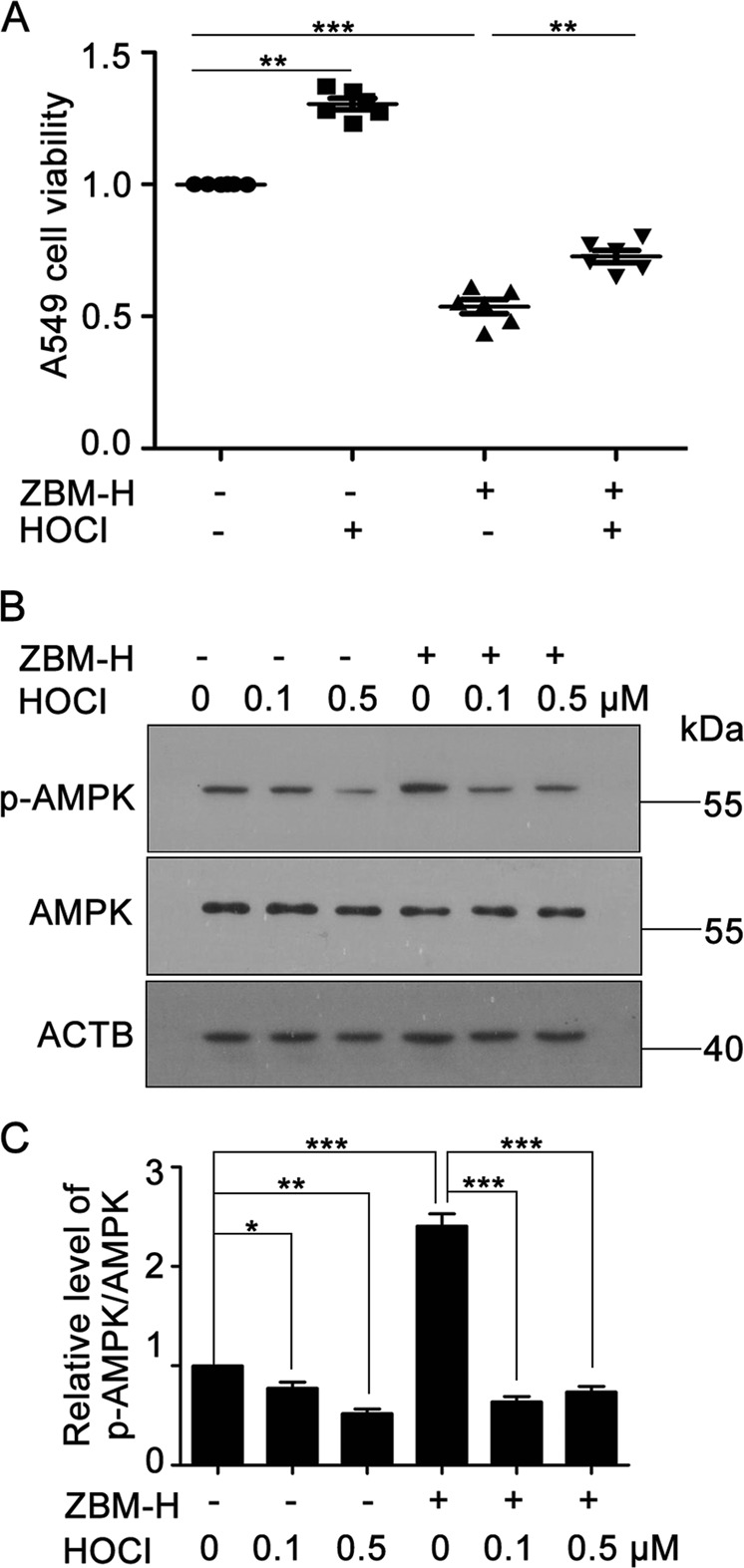
Exogenous hypochlorous acid partially reversed ZBM-H-inhibited cell growth and ZBM-H-increased glucose-regulated protein 78 activity. **a** Cell viability of A549 cells after pretreated with hypochlorous acid (0.1 μM) for 1 h, and then treated with ZBM-H (5 μM) for 12 h*, n* *=* *6*. **b**, **c** Western blot analysis and quantification of phosphorylation of AMPKα (Thr172) after pretreated with hypochlorous acid (0.1, 0.5 μM) for 1 h, and then treated with ZBM-H (5 μM) for 6 h. Data are presented as the mean ± SEM, *p *<* *0.05,* **p *<* *0.01,* ***p *<* *0.001, n* *=* *3*

To further illustrate the specific mechanism by which ZBM-H inhibited tumor growth, we hypothesized that ZBM-H could bind to hypochlorous acid and inhibit the function of hypochlorous acid. The data showed that exogenous hypochlorous acid partially reversed ZBM-H-inhibited cell survival (Fig. 7a). At the same time, the addition of HOCl significantly inhibited the activation of AMPK induced by ZBM-H (Fig. 7b, c). These data indicated that ZBM-H inhibited A549 cell growth via binding to HOCl and inhibiting the function of HOCl.

### ZBM-H inhibited the growth of lung cancer xenografts in the CAM model

**Fig. 8:**
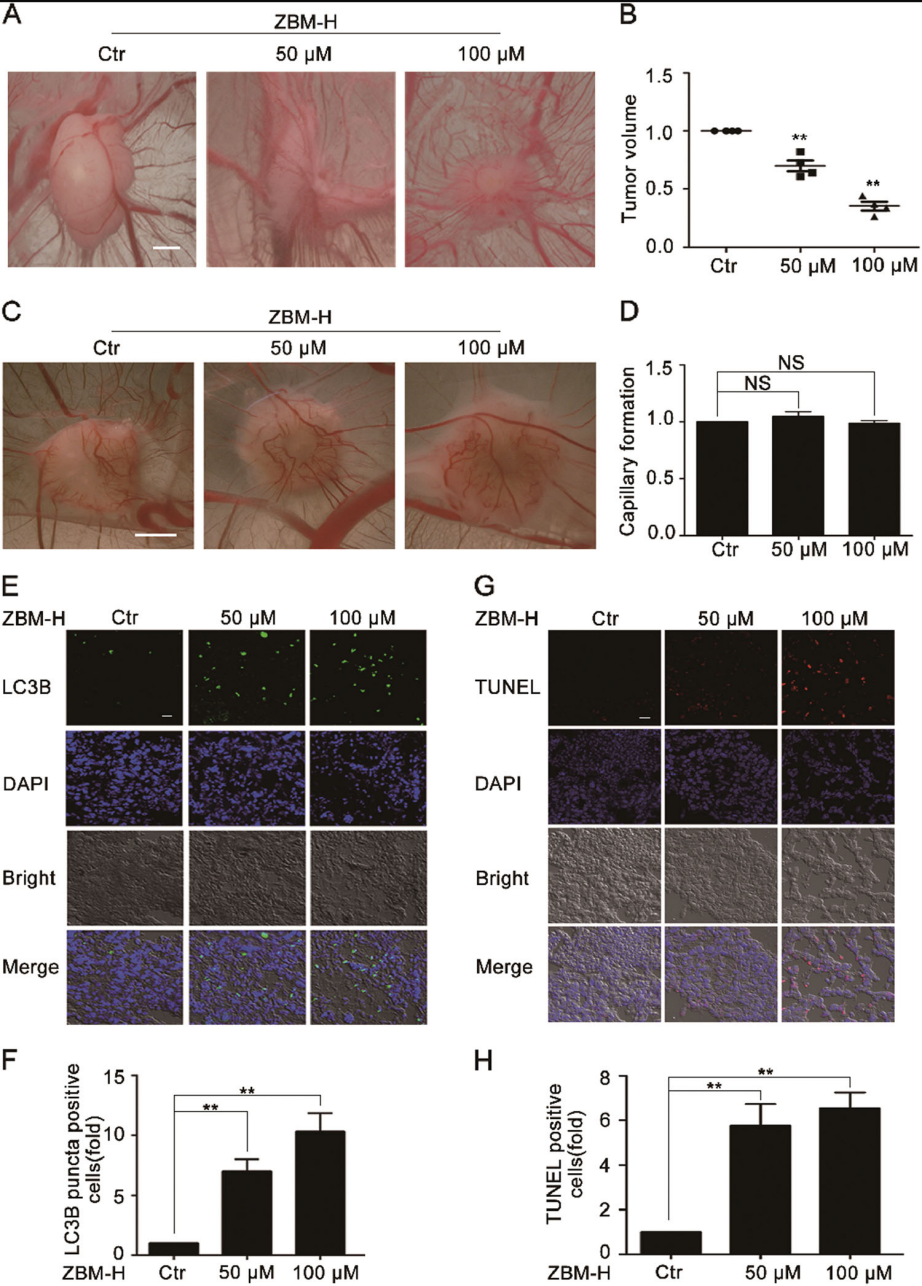
ZBM-H inhibited lung cancer growth in vivo. **a**, **b** Biomicroscopy and quantification of the tumors treated with ZBM-H at indicated concentrations. Scale bar: 1.5 mm. **c**, **d** Biomicroscopy and quantification of angiogenesis on gelatin sponge with ZBM-H adsorption. **e**, **f** Immunohistochemistry (IHC) staining LC3B puncta of the frozen tumors sections. Scale bar: 20 μm. **g**, **h** terminal deoxynucleotidyl transferase-mediated dUTP nick-end labeling (TUNEL) staining of the frozen tumors sections. Scale bar: 20 μm. Data are presented as the mean ± SEM, *NS p* *>* 0.05, **p *<* *0.01*, *n* *=* *3*

A recent study has shown that the disruption of tumor blood vessels normalization effectively promotes tumor migration^36^. In order to better evaluate the anti-tumor effect of ZBM-H in vivo, we chose the chick embryo chorioallantoic membrane (CAM) model for further study. The chick embryo chorioallantoic membrane (CAM) has been increasingly used as the model of tumor engraftments as well as angiogenesis to evaluate the availability of potential anti-cancer drugs since it has immune-deficient environment and the dense capillary network^37^. We firstly investigated the effect of ZBM-H on tumor growth and normal angiogenesis. The results demonstrated that ZBM-H substantially suppressed tumor growth as evidenced by smaller tumor volume of ZBM-H-treated tumors (Fig. 8a, b). We further determined the effect of ZBM-H on normal CAM angiogenesis. Data revealed that ZBM-H had no effect on normal CAM angiogenesis (Fig. 8c, d). Therefore, ZBM-H effectively inhibited tumor growth in vivo without adverse effects on normal CAM angiogenesis.

### ZBM-H inhibited lung cancer growth by inducing autophagy and apoptosis in vivo

To further investigate the mechanism by which ZBM-H inhibited tumor growth in vivo, we prepared frozen sections of solid tumors formed on CAM. Immunofluorescence experiment was performed on frozen sections of tumor. Data showed that ZBM-H induced autophagy in solid tumor, with remarkable LC3B puncta detected (Fig. 8e, f). In addition, we performed TUNEL assay to detect whether ZBM-H could induce cell apoptosis in solid tumor. After the TUNEL staining and confocal microscopy analysis of frozen sections, we discovered that ZBM-H promoted tumor apoptosis significantly in vivo (Fig. 8g, h). These data demonstrated that ZBM-H inhibited lung cancer growth through inducing autophagy and apoptosis in vivo.

## Discussion

Endoplasmic reticulum (ER) is involved in the synthesis of intracellular proteins and lipids and the regulation of calcium homeostasis, thereby playing an important role in cell growth regulation^38^. Although we have previously synthesized and reported many HOCl probes that targeted mitochondria and lysosome, probes targeting endoplasmic reticulum are relatively rare. So far, only one HOCl probe was reported to target endoplasmic reticulum according to literature retrieval^22,23,39–43^. However, the function of HOCl in the endoplasmic reticulum has not been studied. In a previous study we synthesized and identified a new ratiometric fluorescent probe (ZBM-H), which showed good selectivity toward HOCl by analyzing the fluorescence spectra and absorption spectra^24^. In the present study, we found that ZBM-H targeted HOCl in the endoplasmic reticulum. This exciting result made ZBM-H a powerful tool for studying the specific mechanism by which HOCl in the endoplasmic reticulum regulates cell growth. Among those HOCl probes, ZBM-H displays the lowest IC_50_ value in A549 lung cancer cells (Fig. S1). So we first chose ZBM-H for the following research. A large number of probes have been designed to detect HOCl in living cells, which consumed and reacted with HOCl, as evidenced by their new products^2^. Our data revealed that ZBM-H, targeting HOCl in the endoplasmic reticulum, inhibited cell survival. In addition, exogenous HOCl attenuated the effect of ZBM-H on cell growth. These results suggested that ZBM-H inhibited lung cancer cell growth by combining with HOCl.

HOCl is a strong oxidant with high reactivity, which reacts with Cys, Met and Lys of proteins to participate in protein post-translational modifications and regulation of their activity^7–9,44^. GRP78 contains two Cys residues: Cys41 and Cys420, which are located in distinct domains. It has been reported that disulfide bonds between Cys41 and Cys420 residues may be difficult to form by H_2_O_2_ treatment, and oxidized NPGPx would be required to facilitate the process^14^. In this study, the pre-treatment has destroyed the disulfide bond that might form, so it is reasonable that we cannot detect the oxidative modification of cysteine. In addition, GRP78 contains a high abundance of methionine and lysine which can be oxidized and modified. Here, we discovered that oxidation modification of lysine residue of GRP78 has changed. However, oxidation modification of methionine residue of GRP78 might also be found by improving sequence coverage in future studies. As expected, Lys353 mutant compromised ZBM-H-increased GRP78 activity. Here, we reported for the first time about the effect of hypochlorous acid on lysine oxidation modification of GRP78 and its activity in cancer cells, suggesting that GRP78 was potent effector protein involved in HOCl regulating tumor growth.

Autophagy is crucial for the regulation of cancer cell fate^18^. However, it is not clear whether HOCl, especially HOCl in the ER, is involved in the regulation of autophagy. In this study, we first found that ZBM-H, a HOCl probe targeting ER, promoted autophagy by blocking HOCl-induced GRP78 oxidation modification. This provides new evidence for the mechanism of HOCl regulating autophagy in cancer cells. Recently, autophagy has become a novel therapeutic target for cancer treatment^29,45,46^. Increasingly more evidence has shown that autophagy serves a suppressive role in controlling tumor growth through resulting in autophagic cell death or apoptosis, or through inhibiting cell proliferation in an apoptosis-independent manner^16,47^. In this study, we demonstrated that ZBM-H inhibited cell growth by inducing autophagy and apoptosis. In addition, experiments using an autophagy inhibitor 3BDO showed that autophagy inhibition attenuated ZBM-H-induced apoptosis and partially rescued ZBM-H-inhibited cell viability (Fig. S9), suggesting that ZBM-H-induced autophagy was a pro-apoptotic mechanism in A549 cells.

In summary, we found that a hypochlorous acid probe named ZBM-H targeted endoplasmic reticulum. ZBM-H inhibited the Lys 353 oxidation of GRP78 induced by HOCl, thus enhancing the activity of GRP78. Furthermore, ZBM-H effectively inhibited tumor cell growth in vitro and in vivo via stimulating AMPK/mTOR dependent autophagy and apoptosis, thereby providing new evidence for the function of endogenous HOCl-modified proteins regulating cancer cell growth.

## Supplementary information

Supplemental material
