# Supplementary material for: Inhibiting lysine 353 oxidation of GRP78 by a hypochlorous probe targeting endoplasmic reticulum promotes autophagy in cancer cells
Source: Cell Death Dis. 2019 Nov 12;10(11):858. doi: 10.1038/s41419-019-2095-y (PMC6851114; doi:10.1038/s41419-019-2095-y)
Supplement: Supplementary file 1 — Supplemental material [file 41419_2019_2095_MOESM1_ESM.docx]

**Supplementary information**

**Inhibiting lysine 353 oxidation of GRP78 by a hypochlorous probe targeting endoplasmic reticulum promotes autophagy in cancer cells**

Junya Ning^a^, Zhaomin Lin^b^, Xuan Zhao^a^, Baoxiang Zhao^c,*^, Junying Miao^a,d,*^

*^a^Shandong Provincial Key Laboratory of Animal Cells and Developmental Biology, School of Life Science, Shandong University, Qingdao 266237, P.R. China*

*^b^ Central Research Laboratory, the Second Hospital, Shandong University, Jinan,*

*250033, P.R. China*

*^c^ Institute of Organic Chemistry, School of Chemistry and Chemical Engineering, Shandong University, Jinan 250100, P.R. China*

*^d^ The Key Laboratory of Cardiovascular Remodeling and Function Research, Chinese Ministry of Education and Chinese Ministry of Health, Shandong University Qilu Hospital, Jinan, 250012, P.R. China*

*Correspondence to: Prof. Junying Miao and Prof. Baoxiang Zhao, Shandong Provincial Key Laboratory of Animal Cells and Developmental Biology, School of Life Science, Shandong University, Qingdao 266237, P.R. China, Fax: + 86 531 88565610; Tel.: + 86 531 88364929.

E-mail address: [miaojy@sdu.edu.cn](mailto:miaojy@sdu.edu.cn) and bxzhao@sdu.edu.cn


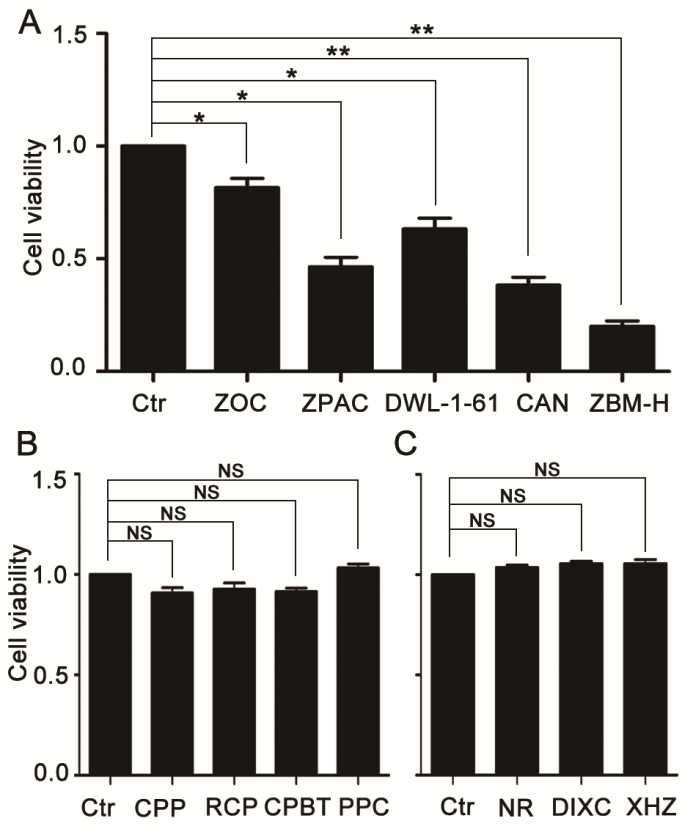


**Figure S1 Effects of small chemical molecules targeting hypochlorous acid on A549 cell viability.** **(A-C)**, A549 cells were treated with indicated small chemical molecules at 5 μM for 24 h, and then the cell viability was determined by SRB assay. **ZOC**,(E)-4-(4-(diethylamino)-2-hydroxystyryl)-1-(2-(4-(6-(diethylamino)-2-oxo-2H-chromene-3-carbonyl)piperazin-1-yl)-2-oxoethyl)pyridin-1-ium;**ZPAC**,(Z)-1-(2-(4-(7-(diethylamino)-2-oxo-2H-chromene-3-carbonyl)piperazin-1-yl)-2-oxoethyl)-4-(4-(dimethylamino)styryl)pyridin-1-ium;**DWL-1-61**,(E)-1-(2,5-dihydroxyphenyl)-3-(1,3-dimethyl-1H-indol-2-yl)prop-2-en-1-one;**CAN**,7-(diethylamino)-3-(2,3-dihydro-1H-perimidin-2-yl)-2H-chromen-2-one;**ZBM-H**,(E)-1,3,3-trimethyl-2-(2-(4-methylbenzo[4,5]imidazo[1,2-a]pyridin-3-yl)vinyl)-3H-indol-1-ium;**CPP**,(E)-4-(4-(4-(7-(diethylamino)-2-oxo-2H-chromene-3-carbonyl)piperazin-1-yl)styryl)-1-methylpyridin-1-ium;**RCP**,4-((3'-(diethylamino)-6'-(4-(7-(diethylamino)-2-oxo-2H-chromene-3-carbonyl)piperazin-1-yl)-3-oxospiro[isoindoline-1,9'-xanthen]-2-yl)carbamoyl)-1-methylpyridin-1-ium;**CPBT**,(E)-2-(4-(4-(7-(diethylamino)-2-oxo-2H-chromene-3-carbonyl)piperazin-1-yl)styryl)-3-methylbenzo[d]thiazol-3-ium;**PPC**,2-((10-(3-(4-(6-(diethylamino)-3-oxo-3,4-dihydronaphthalene-2-carbonyl)piperazin-1-yl)propyl)-4a,10a-dihydro-10H-phenothiazin-3-yl)methylene)malononitrile;**NR**,N-(3',6'-bis(diethylamino)-3-thioxo-4a',9a'-dihydrospiro[isoindoline-1,9'-xanthen]-2-yl)-4-(1,3-dioxo-6-(piperidin-1-yl)-1H-benzo[de]isoquinolin-2(3H)-yl)benzamide;**DIXC**,N-((3',6'-bis(diethylamino)-3-oxospiro[isoindoline-1,9'-xanthen]-2-yl)carbamothioyl)-7-(diethylamino)-2-oxo-2H-chromene-3-carboxamide;**XHZ**,(E)-2-(3-(4-(4-(7-(diethylamino)-2-oxo-2H-chromene-3-carbonyl)piperazin-1-yl)styryl)-5,5-dimethylcyclohex-2-en-1-ylidene)malononitrile. Data are presented as the mean ± SEM, *NS p ＞ 0.05, *p < 0.05, **p < 0.01, n = 3.*

**
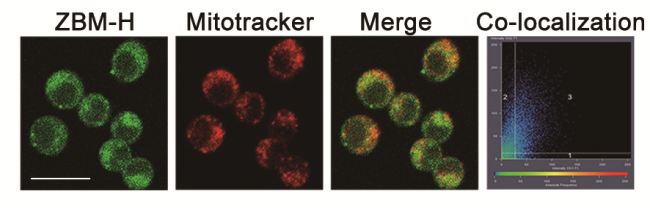
**

Figure S2 ZBM-H had no co-localization with mitochondria. RAW cells were treated with ZBM-H, followed by Mitotracker Deep Red (excitation light of 640 nm) for 30 min, and the co-localization efficiency of ZBM-H and mitochondria was 0.70. Scale bar: 20 μm.

**
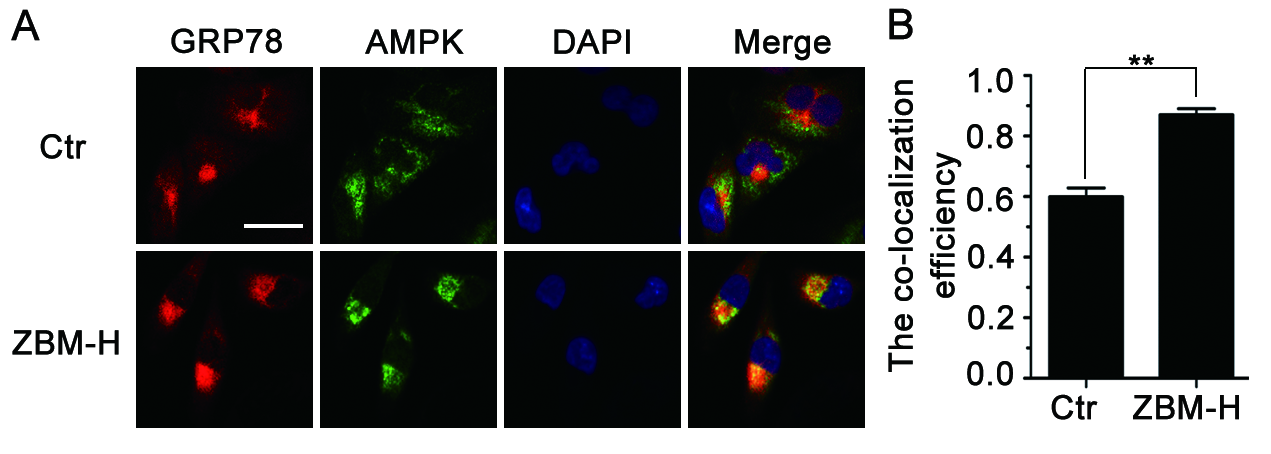
**

Figure S3 Immunoﬂuorescence (IF) assay of the co-localization analysis of GRP78 and AMPKα. (A and B), A549 cells were treated with ZBM-H (2 μM) for 6 h. The fluorescence signals were visualized by confocal microscopy. Scale bar: 20 μm.

**
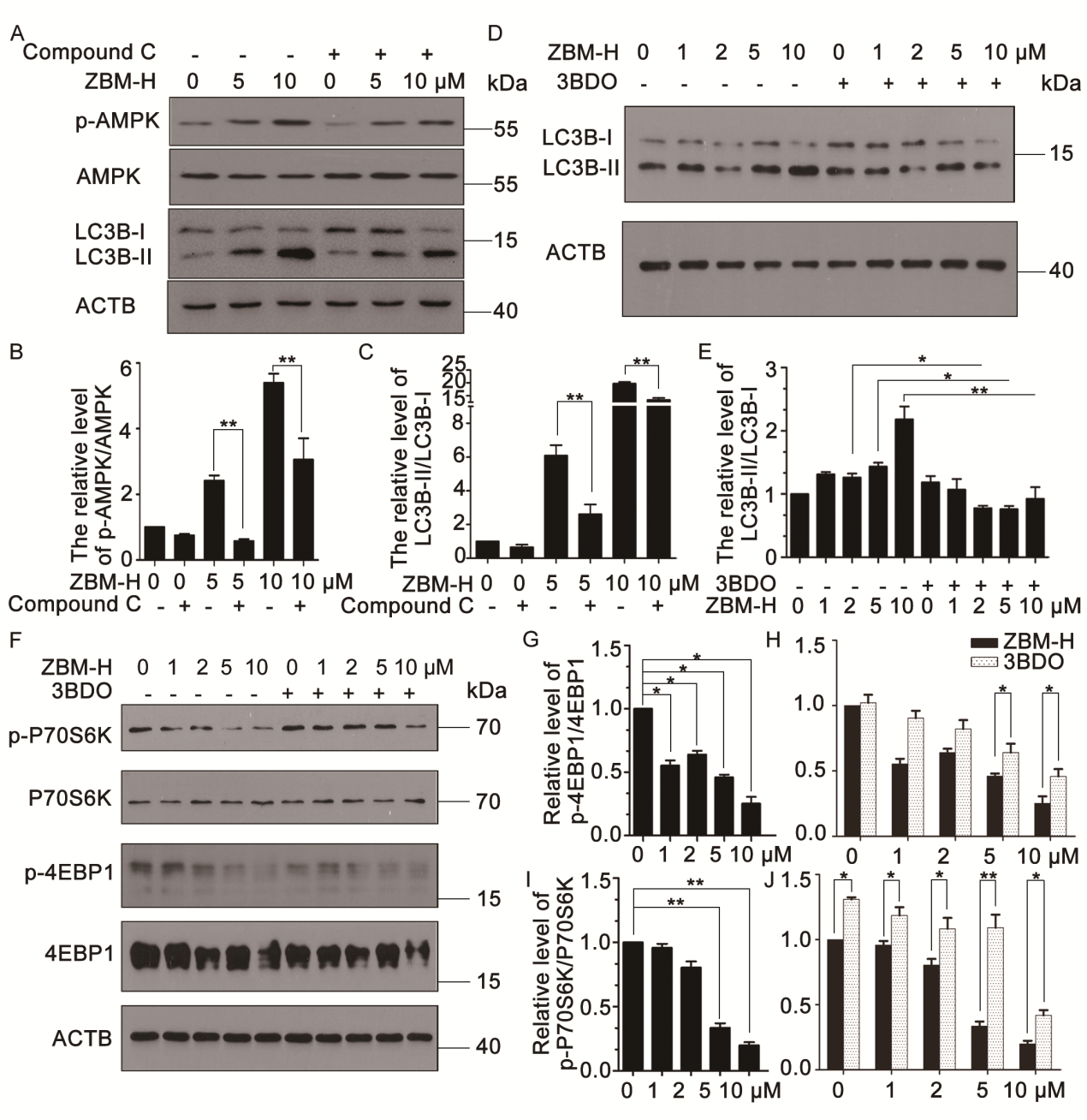
**

Figure S4 AMPK/mTOR pathway was involved in ZBM-H-induced autophagy in lung cancer cells. (A-C), Western Blot analysis of phosphorylation of AMPKα (Thr172) and the level of LC3B-II/LC3B-I in A549 cells pretreated with Compound C for 2 h and treated with ZBM-H at indicated concentrations for 6 h. Western Blot analysis of LC3B-II/LC3B-I (D and E) and p-p70S6K (S424/T421) and p-4EBP1 (S65/T70) (F-J) in A549 cells treated with 3BDO (1 μM) and ZBM-H for 6 h. ACTB was used as loading control. Data are presented as the mean ± SEM, **p < 0.05, **p < 0.01, n = 3.*

**
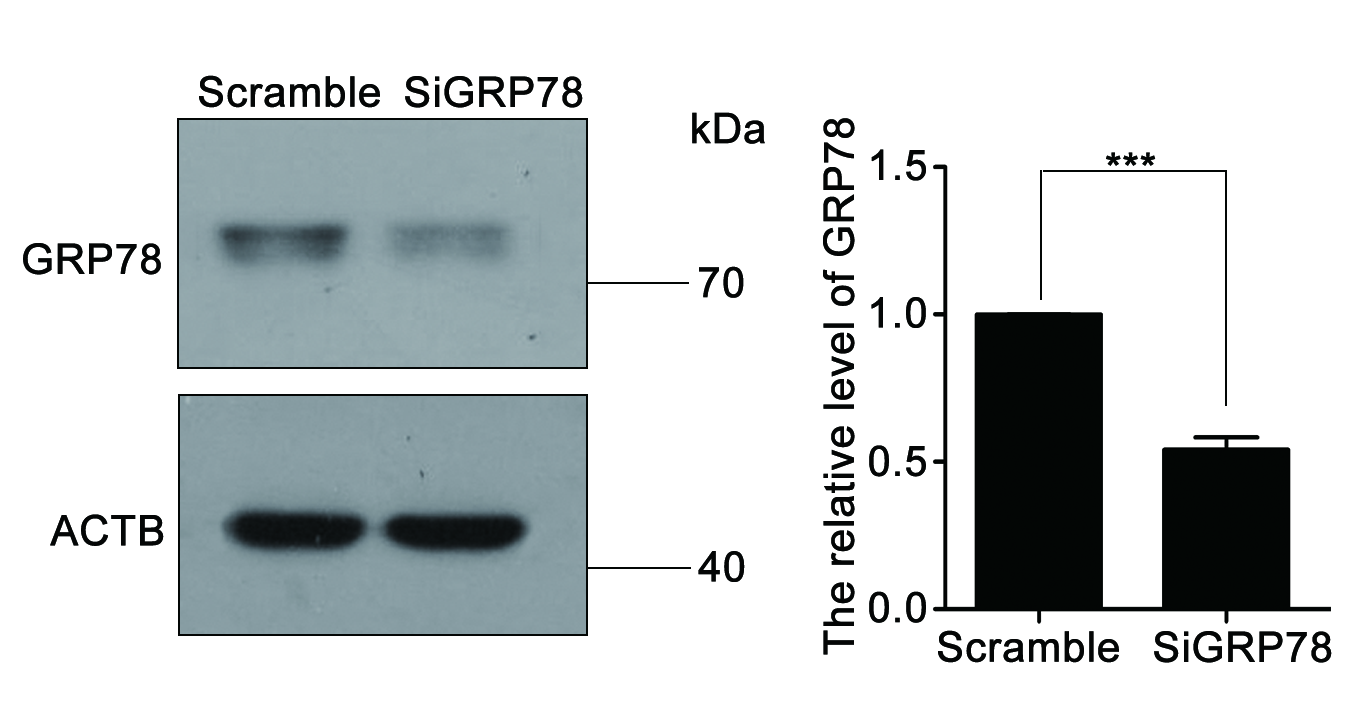
**

Figure S5 Interference efficiency of SiGRP78. WB analysis and quantification of GRP78 in A549 cells transfected with GRP78 siRNA (siGRP78 concentration was 60 nM). Data are presented as the mean ± SEM, ****p < 0.001, n = 3.*

**
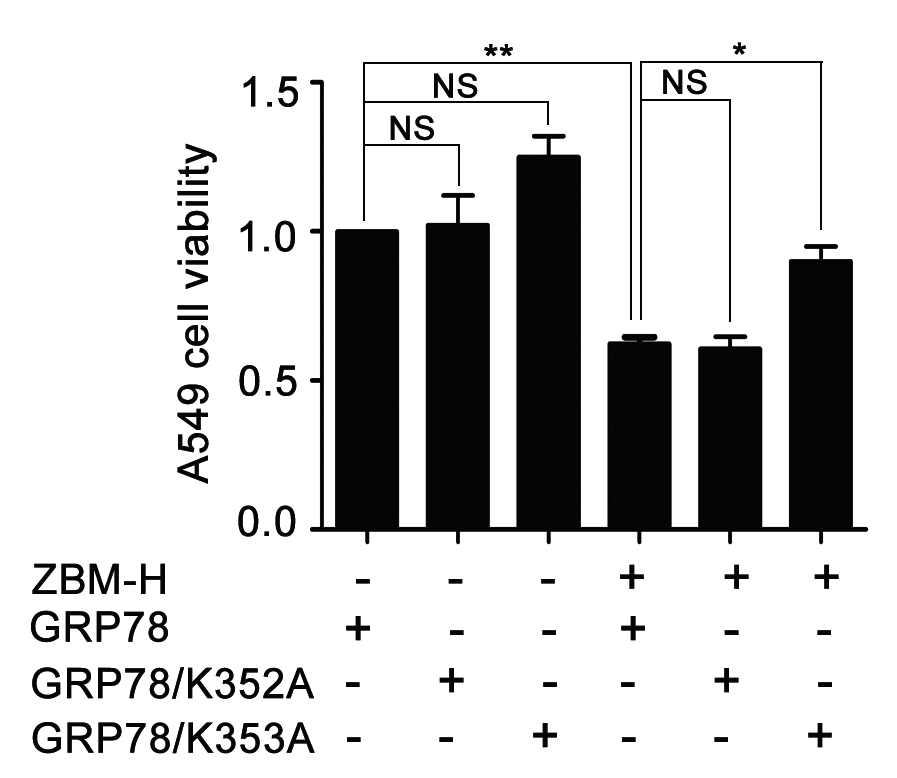
**

Figure S6 A549 cell viability. Viability of A549 cells transfected with his6-GRP78-wt (wild type), his6-GRP78-mut1 (K352A) mutant and his6-GRP78-mut2 (K353A) mutant plasmids and treated with ZBM-H for 24 h. Data are presented as the mean ± SEM, *NS p ＞ 0.05, *p < 0.05, **p < 0.01, n = 3.*

**
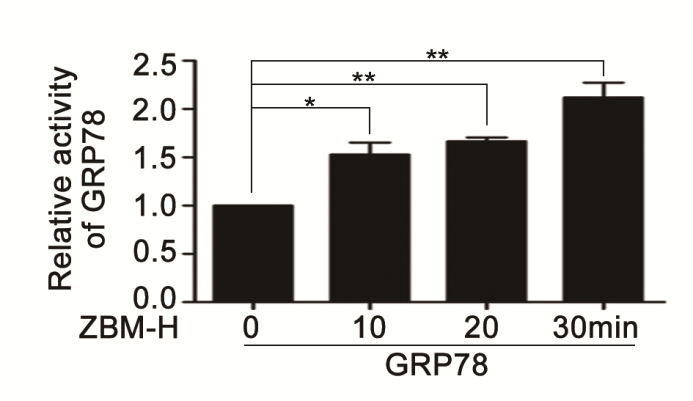
**

Figure S7 ZBM-H enhanced GRP78 activity in a time-dependent manner. ATPase activity of GRP78 incubated with ZBM-H (5 μM) for indicated time. Data are presented as the mean ± SEM, **p < 0.05, **p < 0.01, n = 3*.

**
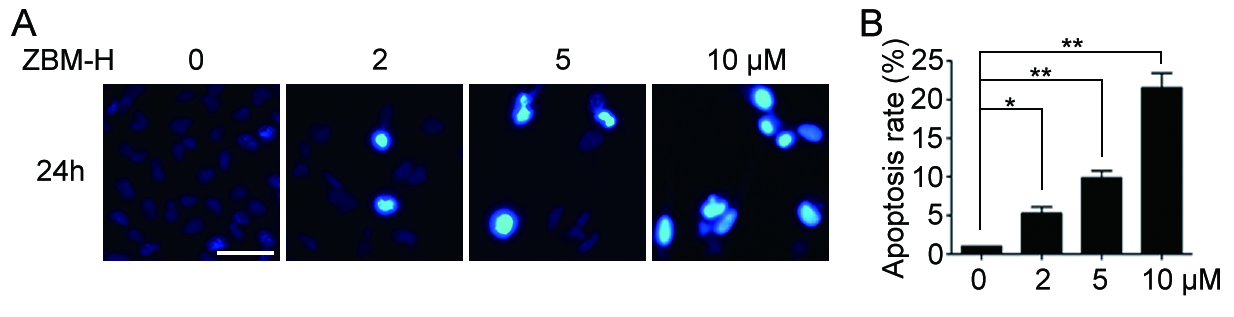
**

Figure S8 Apoptosis of A549 cells treated with ZBM-H. (A and B), Fluorescent images and quantitative statistics of A549 cells treated with ZBM-H at the indicated concentrations for 24 h and stained with Hoechst 33258. Scale bar: 20 μm. Data are presented as the mean ± SEM, **p < 0.05, **p < 0.01, n=3.*

**
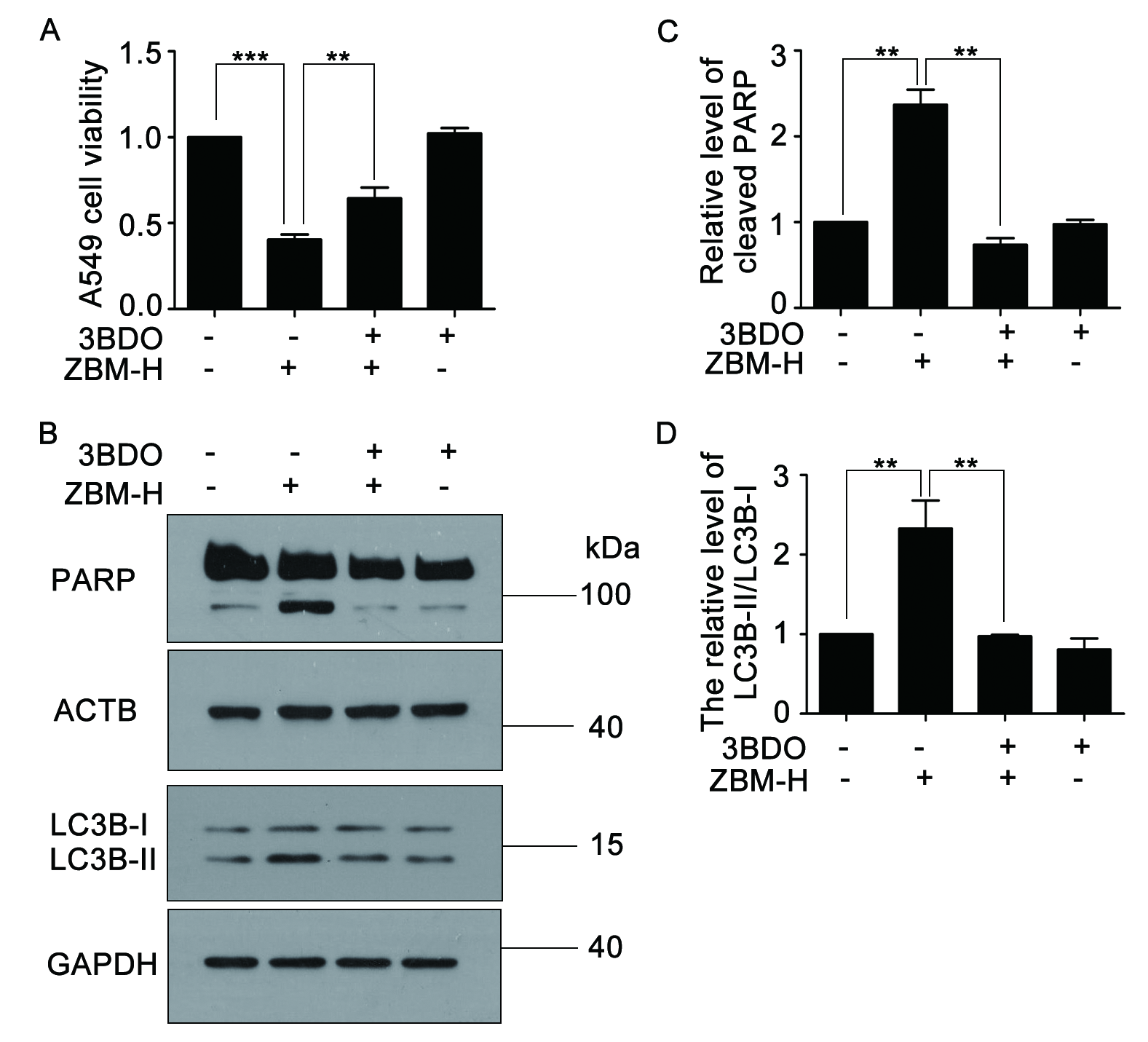
**

Figure S9 3BDO partially rescued ZBM-H-inhibited cell viability and attenuated ZBM-H-induced apoptosis. (A), Viability of A549 cells treated with ZBM-H (5 μM) and 3BDO (1 μM) for 24 h. (B-D), WB analysis and quantification of cleaved poly ADP-ribose polymerase (PARP) and LC3B-II/LC3B-I in A549 cells treated with ZBM-H (5 μM) and 3BDO (1 μM) for 24 h. Data are presented as the mean ± SEM, ***p < 0.01, ***p < 0.001, n = 3.*

**
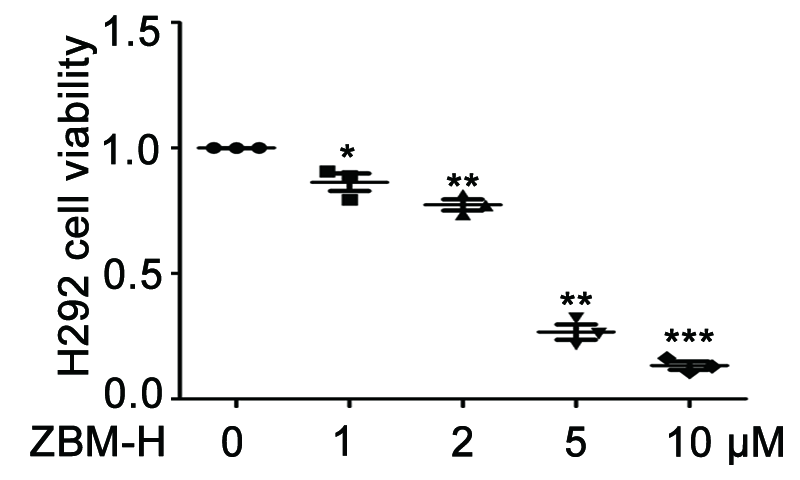
**

Figure S10 H292 cell viability. Viability of H292 cells treated with ZBM-H at indicated concentrations for 24 h. Data are presented as the mean ± SEM, **p < 0.05, **p < 0.01, ***p < 0.001, n = 3.*

Table S1. List of small chemical molecules that targeting hypochlorous acid in Endoplasmic reticulum and cytoplasm.

| Name | Structural formula | Distribution | Cell fate | Published |
| --- | --- | --- | --- | --- |
| ZBM-H | 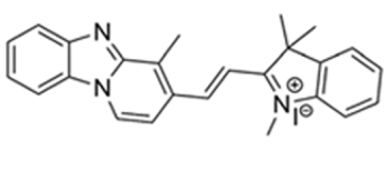 | Endoplasmic reticulum | Inhibiting A549 cell survival  **IC_50_**= ­1.648 ­­­­­μM | DOI: 10.1039/C7NJ03907G |
| CAN | 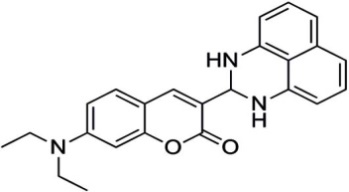 | Cytoplasm | Inhibiting A549 cell survival  **IC_50_**= 3.500 μM | Not yet published |
| ZPAC | 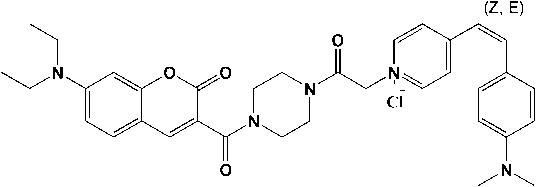 | Cytoplasm | Inhibiting A549 cell survival  **IC_50_**= 4.900 μM | DOI:  10.1016/j.talanta.2019.04.024 |
| DWL-1-61 | 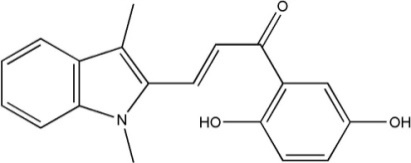 | Cytoplasm | Inhibiting A549 cell survival  **IC_50_**= 7.200 μM | Not yet published |
| ZOC | 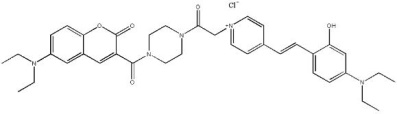 | Cytoplasm | Inhibiting A549 cell survival  **IC_50_**= 14.300 μM | DOI:  10.1016/j.aca.2019.03.004 |

Table S2. List of small chemical molecules that targeting hypochlorous acid in Mitochondria.

| Name | Structural formula | Distribution | Cell fate | Published |
| --- | --- | --- | --- | --- |
| CPP | 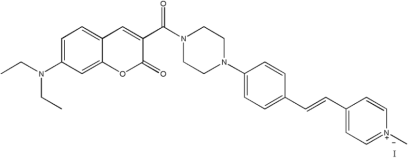 | Mitochondria | No effect on A549 cell survival | DOI:  10.1016/j.snb.2018.08.071 |
| RCP | 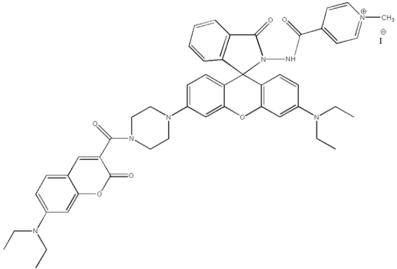 | Mitochondria | No effect on A549 cell survival | DOI:  10.1039.C6TB01992G |
| CPBT | 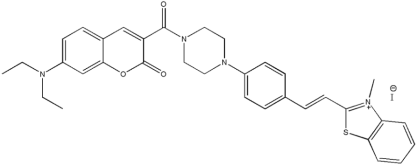 | Mitochondria | No effect on A549 cell survival | DOI：  10.1016/j.aca.2016.11.019 |
| PPC | 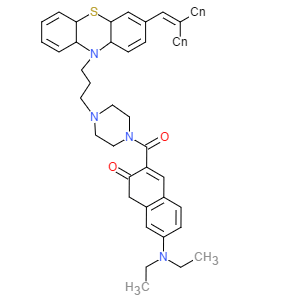 | Mitochondria | No effect on A549 cell survival | DOI：  10.1016/j.dyepig.2019.107708 |

Table S3. List of small chemical molecules that targeting hypochlorous acid in Lysosome.

| Name | Structural formula | Distribution | Cell fate | Published |
| --- | --- | --- | --- | --- |
| NR | 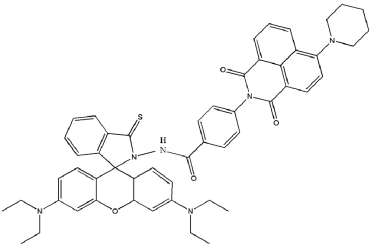 | Lysosome | No effect on A549 cell survival | DOI: 10.1016/j.snb.2017.01.073 |
| DIXC | 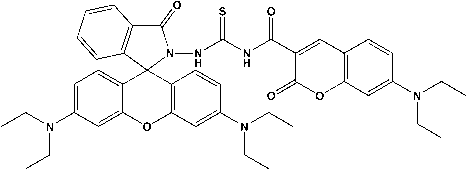 | Lysosome | No effect on A549 cell survival | DOI: 10.1016/j.snb.2017.03.049 |
| XHZ | 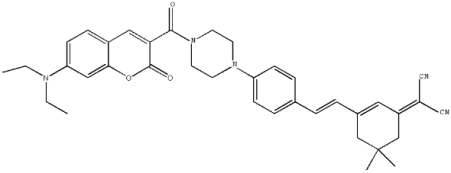 | Lysosome | No effect on A549 cell survival | Not yet published |
